# Copy Number Variation in CNP267 Region May Be Associated with Hip Bone Size

**DOI:** 10.1371/journal.pone.0022035

**Published:** 2011-07-15

**Authors:** Shan-Lin Liu, Shu-Feng Lei, Fang Yang, Xi Li, Rong Liu, Shan Nie, Xiao-Gang Liu, Tie-Lin Yang, Yan Guo, Fei-Yan Deng, Qing Tian, Jian Li, Yao-Zhong Liu, Yong-Jun Liu, Hui Shen, Hong-Wen Deng

**Affiliations:** 1 Laboratory of Molecular and Statistical Genetics and the Key Laboratory of Protein Chemistry and Developmental Biology of Ministry of Education, College of Life Sciences, Hunan Normal University, Changsha, Hunan, People's Republic of China; 2 Center of Bioinformatics and Genomics, School of Public Health and Tropical Medicine, Tulane University, New Orleans, Louisiana, United States of America; 3 School of Life Science and Technology, Xi'an Jiaotong University, Xi'an, Shanxi, People's Republic of China; 4 Center of Systematic Biomedical Research, University of Shanghai for Science and Technology, Shanghai, People's Republic of China; Florida State University, United States of America

## Abstract

Osteoporotic hip fracture (HF) is a serious global public health problem associated with high morbidity and mortality. Hip bone size (BS) has been identified as one of key measurable risk factors for HF, independent of bone mineral density (BMD). Hip BS is highly genetically determined, but genetic factors underlying BS variation are still poorly defined. Here, we performed an initial genome-wide copy number variation (CNV) association analysis for hip BS in 1,627 Chinese Han subjects using Affymetrix GeneChip Human Mapping SNP 6.0 Array and a follow-up replicate study in 2,286 unrelated US Caucasians sample. We found that a copy number polymorphism (CNP267) located at chromosome 2q12.2 was significantly associated with hip BS in both initial Chinese and replicate Caucasian samples with p values of 4.73E-03 and 5.66E-03, respectively. An important candidate gene, four and a half LIM domains 2 (FHL2), was detected at the downstream of CNP267, which plays important roles in bone metabolism by binding to several bone formation regulator, such as insulin-like growth factor-binding protein 5 (IGFBP-5) and androgen receptor (AR). Our findings suggest that CNP267 region may be associated with hip BS which might influence the FHL2 gene downstream.

## Introduction

Osteoporosis is a serious global public health problem especially in the elderly, which is characterized by low bone mineral density (BMD) and low trauma fracture. Osteoporotic hip fracture (HF) is the most serious low trauma fracture with high morbidity and mortality. It has been predicted that the number of osteoporotic fractures (OFs) worldwide will increase to 6.3 million in 2050, with most of these future fractures happening in Asian [1]. The economic burden is estimated at 17 billion dollars every year on OFs since 2005 in the United States alone [2]. Especially in recent years, an increasing aging Chinese population has resulted in even more OFs in China [3].

BMD, as an important risk factor for OFs, has been extensively investigated for identifying the genetic factors underlying BMD variation [4]. However, BMD is not the only risk factor for osteoporotic fracture, and recent studies suggested that bone size (BS) is another important risk factor for OFs independent of BMD [5], [6]. BS has more than 50% of heritability in both Chinese and Caucasians [7], [8]. Abnormal BS contributes significantly to the pathogenesis of OFs [5], [6]. Bigger bone size is a logical adaptation to enhance the mechanical competence of bone, because a larger cross-sectional area can bear larger compressive loads and cope more efficiently with bending loading. Spine BMD in females is comparable with that in males, however, females suffer a larger incidence of spine fractures than males partially attributable to the fact that female's spine bone sizes are 20–25% smaller than male's after adjusting for body size differences [9]. A genetically homogenous inbred mouse strain has higher bone mass but smaller bone size, and is less sensitive in adapting to mechanical loading to increase bone strength when compared with another inbred mouse strain [10]. Currently, only a few of genes such as VDR [11], [12], ER, COL1A2 [13], CYP17 [14], PTH [15], were tested in association with BS, and most of the genetic factors for BS largely remain unknown.

Copy number variation (CNV) is a type of DNA variation due to diversity in the number of copies of a DNA segment that may range from one kilobase to several megabases in size. Copy number polymorphisms (CNPs) refer to common CNVs that appear to involve the same affected genomic sequence and are therefore consistent with a model of a genetic polymorphism. CNVs may alter gene dosage, disrupt coding sequences, or exert long-range positional effects on gene expression pattern outside CNV region, consequently leading to phenotype variation [16], [17]. Individuals with lower copy number of CCL3L1 [18], FCGR3B [19] and DEFB4 [20] genes were predisposed to risk of AIDS, immunologically mediated glomerulonephritis and Crohn disease, respectively. Recently, our groups performed two genome-wide CNV association studies on BMD in Chinese and Caucasian and found CNV regions containing UGT2B17 [21] and VPS13B [22] genes were significantly associated with BMD, respectively. However, to the best of our knowledge, there is no reported genome-wide CNV association research that focused on hip BS variation.

This study performed an initial genome-wide common CNV association analysis in 1,627 Chinese Han subjects using Affymetrix GeneChip Human Mapping SNP 6.0 array and a follow-up replicate study in 2,286 unrelated US Caucasians sample, and identified CNP267 region may be associated with hip BS, which might influence the FHL2 gene downstream.

## Materials and Methods

### Ethics Statement

The study was approved by the necessary institutional review boards of all the participating institutions. Before entering the project, all the subjects signed the informed-consent documents.

### Subjects and Measurement

We first performed an initial genome-wide CNV analysis for hip BS in 1,627 unrelated Chinese Han subjects. The most significant results identified in Chinese sample were further replicated in an unrelated Caucasian sample.

#### 1) Initial study sample

The sample for the initial genome-wide CNV analysis consisted of 1,627 unrelated Chinese Han adults including 825 women and 802 men. All the subjects were recruited from the cities of Xi'an/Changsha and their vicinity with the purposes of searching for genetic factors for osteoporosis related phenotypes (such as BMD, BS, bone geometry). In order to minimize the confounding effects of environmental and therapeutic factors that may influence the skeletal system, individuals with chronic diseases and conditions that might potentially affect bone metabolism were excluded. These diseases/conditions included chronic disorders involving vital organs (heart, lung, liver, kidney, and brain), serious metabolic diseases (diabetes, hypo- and hyper-parathyroidism, etc.), other skeletal diseases (Paget disease, osteogenesis imperfecta, rheumatoid arthritis, etc.), chronic use of drugs affecting bone metabolism (hormone replacement therapy, corticosteroid therapy, anti-convulsant drugs), and malnutrition conditions (such as chronic diarrhea, chronic ulcerative colitis, etc.), etc. In addition, subjects taking anti-bone-resorptive or bone anabolic agents/drugs, such as bisphosphonates, were excluded from this study.

Hip BS (total area including femoral neck, trochanter and intertrochanter) was measured by a Hologic QDR 4500W dual-energy X-ray absorptiometry (DXA) scanner (Hologic Corp., Waltham, MA). The machine was calibrated daily, and the coefficient of variation (CV) for hip BS value, obtained from seven individuals repeatedly measured five times, of the DXA measurements was 1.18%. Height and weight were measured at the same visit for BS measurement.

#### 2) Caucasian replication sample

To replicate the associations for CNPs at significant level of p<0.01, we performed another independent study in a sample including a total of 2,286 random Caucasian subjects (age: 51.4±13.8 yrs) that were recruited in Midwestern US in Kansas City, Missouri and Omaha, Nebraska for osteoporosis study. All identified subjects were US Caucasians of European origin. The BS values of total hip were measured using DXA machines (Hologic Inc., Bedford, MA, USA) that were calibrated daily. The CV of the DXA measurement for total hip BS was about 1.94%.

### Genotyping

#### 1) In initial study sample

Genomic DNA was extracted from peripheral blood leukocytes using standard protocols. Genotyping with Affymetrix Genome-Wide Human SNP Array 6.0, which features 1.8 million genetic markers, including more than 906,600 SNPs and more than 946,000 probes for detection of copy number variation, was performed using the standard protocol recommended by the manufacturer. Fluorescence intensities were quantified using an Affymetrix array scanner 30007G. Data management and analyses were performed using the Affymetrix® GeneChip® Command Console® Software (AGCC). Contrast quality control (QC) threshold was set at the default value of greater than 0.4 for sample quality control. The final average contrast QC across the entire sample reached the high level of 2.62. The Birdsuite package (http://www.broadinstitute.org/science/programs/medical-and-population-genetics/birdsuite/birdsuite-0) was used for genotype calling, genotyping quality control, and CNV identification.

For the QC for sample, we firstly measured the copy number estimates of each chromosome and genome-wide average (sum of all chromosomes), reported by the Birdseye Hidden Markov Model [23], and removed the subjects who showed excessively high or low estimate for copy number according to either genome-wide average or more than 2 chromosomes (>3 standard deviations). Then, we measured the variability of CNP and SNP probe intensities according to each chromosome and genome-wide average (sum of all chromosomes). We removed the subjects with excessive variability in probe intensity according to either genome-wide average or more than 2 chromosomes (>3 standard deviations). We kept the subjects who only had 1 or 2 chromosomes failing in copy number estimate QC and probe intensity QC, and treated the CNPs in the chromosomes of those subjects as missing data in further association analysis. As a result, 1,531 samples were used in CANARY software [23] for CNP call.

For the QC for CNPs, we removed the CNPs with more than 5% of uncertain or missing copy call (CC) or with less than 1% of allele frequency (AF) that refers to the total proportion of the subjects with copy number less or more than two in total sample. Of the initial full-set of 1,316 CNPs, there are 817 CNPs with AF <1%, 735 CNPs with CC greater than 0.05 and 430 CNPs with both. Finally 194 CNPs covering ∼12 Mb with an average marker spacing of 64 kb remain in the final association analyses (Table S1).

#### 2) In replication sample

Genomic DNA was extracted from human blood using a commercial isolation kit (Gentra systems, Minneapolis, MN, USA) following the protocols detailed in the kit. Genotyping and quality control procedures are the same as in Chinese.

### Statistical analysis

We used stepwise regression model to screen significant covariates for each study cohort. Parameters including age, age^2^, sex, age-sex, age^2^-sex, weight, height, BMI, birth year were tested for their association with hip BS. Significant (p≤0.05) parameters (weight, height in initial Chinese sample and age, sex, age-sex, age^2^-sex, height, birth year in replication Caucasian sample) were then included as covariates to adjust the raw BS values. EIGENSTRAT was employed to perform principal component analysis to correct for stratification in genome-wide association studies [24]. We used ∼370,000 SNPs to calculate the principal components and the ten default main eigenvectors were used as covariates to adjust the raw BS values for correction of population stratification. The adjusted BS data, if not following normal distributions, were further subjected to BoxCox transformation into normal distribution. Finally, association analyses between CNPs and hip BS were performed using the PLINK software package (version 1.07) (http://pngu.mgh.harvard.edu/~purcell/plink/).

The CNPs at the significant level of p<0.01 identified in Chinese sample were selected in replication study in Caucasian. The processes of raw hip BS value adjustment and association analyses in replication study were the same as in initial discovery study in Chinese.

## Results

The basic characteristics of the subjects used in association analyses and replication analyses, including sex, age, height, weight, hip BS were summarized in Table 1.

**Table 1:** Basic characteristics of the study subjects.

Trait	Initial Chinese sample			Caucasian replication sample		
	Total (N = 1,627)	Male (N = 802)	Female (N = 825)	Total(N = 2281)	Male(N = 555)	Female(N = 1726)
Age(year)	34.45 (13.23)	31.43 (11.93)	37.39 (13.76)	51.33(13.74)	50.62(16.04)	51.56(12.91)
Height(cm)	164.25 (8.16)	170.28 (5.95)	158.38 (5.22)	166.35(8.47)	175.87(7.24)	163.29(6.27)
Weight(kg)	60.09 (10.48)	65.72 (9.63)	54.61 (8.09)	75.26(17.53)	87.08(16.72)	71.46(16.04)
Hip BS (cm^2^)	34.05 (5.71)	38.01 (4.01)	30.20 (4.30)	38.35(6.37)	45.86(5.84)	35.93(4.32)

Note: Presented as means (SD).

In the initial genome-wide CNV analysis in Chinese sample, four CNPs were associated with hip BS at significant level of p<0.01 (CNP11164 P = 6.18E-04, CNP182 P = 1.05E-03, CNP267 P = 4.73E-03, CNP10799 P = 7.23E-03) (Table 2). To find replication association evidence, we performed a subsequent replication analysis for these four CNPs in Caucasians that have evidently different genetic background from Chinese. Due to AF of CNP11164 (AF = 0.0031) and CNP10799 (AF = 0.003) is smaller than 1%, only CNP182 and CNP267 were analyzed for replicate association and reached p values at 2.39E-01 and 5.66E-03, respectively (Table 2). Meta-analysis (Fisher's combined *p* method [25]) was used to combine association tests in the two populations. The Fisher's combined p value (p = 3.09E-04) for CNP267 is nearly approaching the significant level after multiple testing (0.05/194 = 2.58E-04).

**Table 2:** Characteristics of the interesting CNPs for association analysis.

NAME	Chr	Start	End	Initial Chinese sample			Caucasian replication sample			Combined P-value
				P value	AF	CC	P value	AF	CC	
**CNP267**	**2**	**106247145**	**106251789**	**4.73E-03**	**0.6328**	**0.0129**	**5.66E-03**	**0.5549**	**0.0182**	**3.09 E-04**
CNP182	1	246815817	246863836	1.05E-03	0.3055	0.0030	2.39E-01	0.2686	0.0053	**2.33 E-03**
CNP11164	6	162658558	162660430	6.18E-04	0.1081	0.0441	N/A	0.0031	0.0364	N/A
CNP10799	4	138543995	138549443	7.23E-03	0.0103	0.0478	N/A	0.0030	0.0205	N/A

Note:

1. AF: allele frequency is calculated as the total proportion of subjects with copy number less or more than two in total samples; CC: uncertain or missing copy calls of CNPs; N/A:not available.

2. This table only lists the association information for 4 interesting CNPs with p<0.01 in the initial genome-wide CNV association analysis, and the information for other 190 CNPs with p>0.01 was presented in the Table S1. Due to the low AF of last two CNPs in replication sample, no association analysis was performed.

3. Combined *p* value: Fisher's combined *p* method [25] was used to combine association tests in the two populations.

4. The NCBI reference genome is Bulid 36.1.

**Figure 1:**
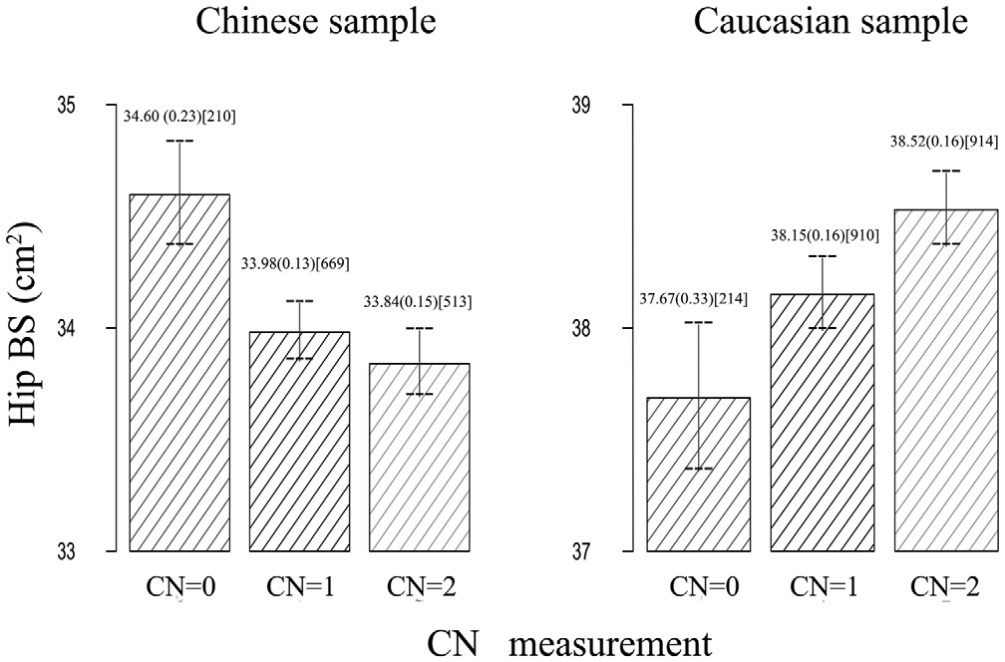
Hip BS values in groups with different copy number (CN) of CNP267 in the Chinese and Caucasians. Data presented are adjusted mean (SE: standard error) [observation number]; CN: copy number.

CNP267 was located in 2q12.2 with physical position from 106,247,145 bp to 106,251,789 bp. The four and a half LIM domains 2 (FHL2) located at the downstream of CNP267, is an important candidate gene, which plays important roles in bone metabolism by binding to several bone formation regulator (details in Discussion). Of the subjects analyzed in the initial and replication samples, carriers of copy number (CN) of 0CN, 1CN, 2CN and 3CN, were 210, 669, 513, 5 in Chinese sample and 214, 910, 914, 7 in Caucasian sample, respectively. Cause the number of carriers of CN = 3 is too small to be statistic significant, we excluded the data of CN = 3 in the following analysis. For the effect of CNP267, we compared the adjusted hip BS for subjects with different CNs of CNP267 in both the initial and replication studies. As shown in Figure 1, CN was negatively associated with hip BS in Chinese sample (i.e., individuals with more CNs trend to have lower hip BS), but positively in Caucasian sample. The opposite effect of CN on BS variation may be caused by several reasons (details in Discussion). Regression analysis showed that CNP267 can explain ∼0.97% of hip BS variation.

## Discussion

CNV is a type of recently increasingly being recognized DNA variation associated with human complex diseases. CNV presumably alters gene dosage, disrupts coding sequences, or exerts long-range positional effects on the gene expression pattern outside the CNV region, and consequently leads to phenotype variation. We performed the first genome-wide CNV association study in a large Chinese sample for an important bone strength phenotype, hip BS, and a follow-up replicate study in 2,286 unrelated US Caucasians sample, and identified that CNP267 region may be associated with hip BS. We also tried to find if there are any BS candidate genes in the regions of significant CNPs in initial Chinese sample. Although there were no well known BS candidate genes, such as VDR, ER, COL1A2, CYP17, PTH et al., we suggested that these genes should deserve more attention in further studies and presented the results in Table S2.

CNP267 was located in 2q12.2 with physical position from 106,247,145 bp to 106,251,789 bp, where no important candidate gene has known function directly associated with osteoporosis or bone. However in the downstream of CNP267 there is an important candidate gene, the four and a half LIM domains 2 (FHL2), which plays important roles in bone metabolism by binding to several bone formation regulator. FHL2 is strongly expressed in all types of human osteoblasts [26]. FHL2 can bind to several regulation factors that are important during bone formation. For example, it binds to insulin-like growth factor-binding protein 5 (IGFBP-5) [26], the most abundant IGFBP stored in bone, which plays an important role in regulating bone formation [27], [28], [29], [30]. FHL2 also increases transcriptional activity of androgen receptor (AR) [31], which is important in regulation of bone formation and resorption [32], [33]. In addition, FHL2 can also influence bone formation as one of modulators of the Wnt signaling pathway [34], [35] and TNF receptor–associated factor 6 (TRAF6)-mediated receptor activator of NF-κB (RANK) signaling pathway [36]. Gene knockdown studies showed that FHL2-deficient mice have decreased BMD and BS due to decreased osteoblast activity [37], [38]. Conversely, enforced expression of FHL2 boosts mineralization in cell culture and increased bone mass in transgenic mice [37].

Genes located outside of CNV intervals can show disrupted expression patterns by long-range position effects. For example, transcription levels of some genes located in the diploid flanking sequence outside deletion region were altered in patients carrying the Williams-Beuren syndrome deletion [39]. CNV17p11.2 can change the expression of some flanking genes, and thus cause Smith-Magenis syndrome [40]. Whole genome association studies between CNVs and gene expression levels showed that more than half of identified genes significantly associated with CNVs were not located in the CNV intervals [41]. These observations may change the traditional view, i.e., CNVs regulate gene expression via directly altering copy number of dosage-sensitive genes located in CNV intervals. However CNVs may alter the expression of genes located outside of CNV intervals by disrupting local chromatin environment and/or gene regulatory elements such as enhancers, silencers, and insulators. Previous studies reported that the relative expression levels of genes were modified through changes in copy numbers as far as 2–7 Mb away from the breakpoints [41], [42].

In association study, the inconsistence of association directions between initial and replicate association studies is usually observed. There may be several reasons to address the opposite effect of CN on BS variation. First, it is well known that the same risk locus with diverse genetic background may generate different effects [43], [44]. Studies also showed that osteoporosis related phenotypes are clearly under ethnic specific genetic determination [45], [46], [47]. Chinese and Caucasians are two evidently different populations in genetic background. The comparison of frequencies of CNs in the two samples shows significantly different with P<0.01 in chi-square test. The ethnic difference may lead to the reverse effect of association in our study. Second, in most situations, significant association suggests strong linkage disequilibrium between marker and causal locus. If the causal loci are different in two samples, it is possible to cause discordant direction of association. For example, the risk allele of marker is negatively correlated with a risk allele at one causal locus in one population, but positively associated with a protective allele at another causal locus in another population. Czarnomska et al. also reported such observation, i.e., a set of genes control the impact of the ApcMin mutation in two organs but with opposite effects [48]. Finally, environmental covariates may influence the effect direction of gene variants. Eder et al. discovered opposite effects of CD14/−260 on serum IgE levels in children raised in different environments [49]. Reneland et al. found that rs1498608 in PDE4D gene showed an opposite relationship with BMD variation, which indicating that the variant's effect may be context-dependent [50]. The two samples come from different geographic regions, suggesting obviously different environment such as living condition and dietary habit. These differences may also influence the effect direction of the allele.

In this study, FHL2 may be recognized as an important candidate gene involved in the association between CNP267 and hip BS by the potential mechanism that CNP267 may influence the expression of FHL2 by causing changes in local chromatin structure or alterations in enhancer activity. This potential mechanism is waiting for being confirmed by functional studies.

In conclusion, this is the first genome-wide CNV association study for hip BS in Chinese Han sample. CNP267 was significantly associated with hip BS in both Chinese and Caucasian. Our findings suggest that CNP267 region may be associated with hip BS.

## Supporting Information

Table S1 **Information of other 190 CNPs with p>0.01 in initial Chinese association analysis.**
(DOC)

Table S2 **Candidate genes at the interesting CNP regions in initial Chinese sample.**
(DOC)
