# Supplementary material for: Copy Number Variation in CNP267 Region May Be Associated with Hip Bone Size
Source: PLoS One. 2011 Jul 15;6(7):e22035. doi: 10.1371/journal.pone.0022035 (PMC3137628; doi:10.1371/journal.pone.0022035)
Supplement: Table S1 — Information of other 190 CNPs with p>0.01 in initial Chinese association analysis. (DOC) [file pone.0022035.s001.doc]

**Table S1. Information of other 190 CNPs with p>0.01 in initial Chinese association analysis**

| NAME | Chr | Start | End | P value | AF | CC |
| --- | --- | --- | --- | --- | --- | --- |
| CNP1196 | 7 | 154024104 | 154031766 | 1.28E-02 | 0.9088 | 0.0387 |
| CNP1222 | 8 | 3774468 | 3777514 | 1.94E-02 | 0.0858 | 0.0304 |
| CNP930 | 6 | 31394255 | 31404430 | 3.27E-02 | 0.8070 | 0.0281 |
| CNP168 | 1 | 227883479 | 227886819 | 3.75E-02 | 0.7177 | 0.0312 |
| CNP1811 | 12 | 9524645 | 9619559 | 4.28E-02 | 0.6811 | 0.0015 |
| CNP12616 | 18 | 74763700 | 74765854 | 4.63E-02 | 0.0191 | 0.0433 |
| CNP147 | 1 | 194997658 | 195068695 | 5.86E-02 | 0.8846 | 0.0167 |
| CNP11858 | 11 | 34177080 | 34179954 | 6.05E-02 | 0.9832 | 0.0334 |
| CNP11931 | 11 | 123592514 | 123601126 | 6.24E-02 | 0.9772 | 0.0426 |
| CNP2430 | 19 | 56834427 | 56840009 | 6.54E-02 | 0.1479 | 0.0296 |
| CNP1984 | 14 | 20419446 | 20479821 | 6.66E-02 | 0.9893 | 0.0114 |
| CNP668 | 4 | 122501918 | 122504766 | 6.85E-02 | 0.7851 | 0.0220 |
| CNP1434 | 9 | 43255666 | 43735571 | 1.05E-01 | 0.5636 | 0.0403 |
| CNP1119 | 7 | 90869671 | 90878663 | 1.07E-01 | 0.6405 | 0.0213 |
| CNP1103 | 7 | 70058925 | 70064077 | 1.15E-01 | 0.0844 | 0.0251 |
| CNP2113 | 15 | 74678296 | 74682830 | 1.20E-01 | 0.4854 | 0.0395 |
| CNP1174 | 7 | 141416112 | 141438377 | 1.30E-01 | 0.7087 | 0.0076 |
| CNP399 | 3 | 37957108 | 37961932 | 1.38E-01 | 0.8688 | 0.0023 |
| CNP11562 | 9 | 11931913 | 11942799 | 1.48E-01 | 0.9796 | 0.0357 |
| CNP2349 | 18 | 63110203 | 63118245 | 1.48E-01 | 0.4779 | 0.0106 |
| CNP1836 | 12 | 33192673 | 33198641 | 1.49E-01 | 0.7725 | 0.0281 |
| CNP10734 | 4 | 71262854 | 71283637 | 1.49E-01 | 0.9772 | 0.0456 |
| CNP12798 | 22 | 20716434 | 20721593 | 1.53E-01 | 0.9872 | 0.0091 |
| CNP35 | 1 | 34875241 | 34877078 | 1.57E-01 | 0.8071 | 0.0205 |
| CNP10964 | 5 | 114734342 | 114748984 | 1.66E-01 | 0.9706 | 0.0129 |
| CNP12463 | 16 | 75758984 | 75780371 | 1.94E-01 | 0.9470 | 0.0289 |
| CNP2384 | 19 | 20388046 | 20513077 | 1.98E-01 | 0.8132 | 0.0008 |
| CNP977 | 6 | 79025784 | 79091904 | 1.98E-01 | 0.9471 | 0.0441 |
| CNP10127 | 1 | 146780429 | 147056085 | 2.05E-01 | 0.9720 | 0.0464 |
| CNP11150 | 6 | 154163673 | 154169933 | 2.05E-01 | 0.9809 | 0.0418 |
| CNP10277 | 2 | 40780879 | 40803110 | 2.06E-01 | 0.9652 | 0.0281 |
| CNP11904 | 11 | 81189919 | 81194913 | 2.14E-01 | 0.9318 | 0.0418 |
| CNP10324 | 2 | 87668332 | 87753000 | 2.20E-01 | 0.9701 | 0.0334 |
| CNP1284 | 8 | 25030439 | 25040250 | 2.20E-01 | 0.6190 | 0.0167 |
| CNP1404 | 9 | 11957033 | 11965492 | 2.21E-01 | 0.9705 | 0.0479 |
| CNP530 | 3 | 190846372 | 190847332 | 2.27E-01 | 0.3968 | 0.0471 |
| CNP12309 | 15 | 31693757 | 31696262 | 2.28E-01 | 0.9652 | 0.0258 |
| CNP1162 | 7 | 133435735 | 133449694 | 2.29E-01 | 0.5944 | 0.0243 |
| CNP10181 | 1 | 188236989 | 188253049 | 2.33E-01 | 0.9899 | 0.0258 |
| CNP11043 | 6 | 31467630 | 31559455 | 2.35E-01 | 0.9548 | 0.0152 |
| CNP12010 | 12 | 69785308 | 69796522 | 2.49E-01 | 0.9489 | 0.0372 |
| CNP2070 | 15 | 28235887 | 28289587 | 2.49E-01 | 0.9860 | 0.0471 |
| CNP2493 | 20 | 45213513 | 45222336 | 2.55E-01 | 0.8673 | 0.0038 |
| CNP703 | 4 | 156022455 | 156022528 | 2.57E-01 | 0.2518 | 0.0410 |
| CNP1245 | 8 | 7891705 | 7903572 | 2.57E-01 | 0.9813 | 0.0198 |
| CNP12017 | 12 | 83237406 | 83241893 | 2.59E-01 | 0.9680 | 0.0342 |
| CNP10681 | 4 | 25323324 | 25339974 | 2.68E-01 | 0.9804 | 0.0327 |
| CNP223 | 2 | 52605074 | 52635046 | 2.74E-01 | 0.1878 | 0.0144 |
| CNP1395 | 9 | 4518770 | 4519873 | 2.76E-01 | 0.9351 | 0.0228 |
| CNP10062 | 1 | 47486248 | 47501675 | 2.80E-01 | 0.9784 | 0.0251 |
| CNP1744 | 11 | 67396131 | 67419052 | 2.90E-01 | 0.9137 | 0.0084 |
| CNP10709 | 4 | 57951132 | 57953111 | 2.99E-01 | 0.7948 | 0.0479 |
| CNP2576 | 22 | 37693565 | 37705253 | 3.08E-01 | 0.4433 | 0.0167 |
| CNP248 | 2 | 87227404 | 87267503 | 3.10E-01 | 0.9764 | 0.0099 |
| CNP504 | 3 | 163699323 | 163709653 | 3.18E-01 | 0.6700 | 0.0182 |
| CNP1586 | 10 | 47012100 | 47165567 | 3.20E-01 | 0.9650 | 0.0319 |
| CNP1902 | 12 | 130382166 | 130391707 | 3.29E-01 | 0.9663 | 0.0403 |
| CNP2529 | 21 | 43794765 | 43797240 | 3.38E-01 | 0.0737 | 0.0129 |
| CNP2735 | 24 | 24377178 | 26584649 | 3.43E-01 | 0.2063 | 0.0152 |
| CNP1675 | 11 | 4924689 | 4933658 | 3.65E-01 | 0.4283 | 0.0342 |
| CNP554 | 4 | 9823254 | 9844366 | 3.69E-01 | 0.2145 | 0.0296 |
| CNP1416 | 9 | 23353115 | 23363484 | 3.71E-01 | 0.2350 | 0.0091 |
| CNP1293 | 8 | 39354760 | 39506122 | 3.75E-01 | 0.7133 | 0.0395 |
| CNP122 | 1 | 167500598 | 167508390 | 3.82E-01 | 0.7727 | 0.0441 |
| CNP28 | 1 | 25465715 | 25534592 | 3.84E-01 | 0.8941 | 0.0190 |
| CNP1796 | 12 | 739370 | 744290 | 3.90E-01 | 0.9035 | 0.0198 |
| CNP207 | 2 | 34552819 | 34590561 | 3.92E-01 | 0.9428 | 0.0114 |
| CNP11545 | 9 | 5301567 | 5327707 | 3.98E-01 | 0.9662 | 0.0205 |
| CNP10118 | 1 | 111629849 | 111637021 | 4.00E-01 | 0.9742 | 0.0182 |
| CNP1732 | 11 | 55130608 | 55209585 | 4.01E-01 | 0.3851 | 0.0023 |
| CNP10138 | 1 | 149603323 | 149664502 | 4.02E-01 | 0.9837 | 0.0030 |
| CNP1107 | 7 | 76163693 | 76388138 | 4.15E-01 | 0.9900 | 0.0137 |
| CNP1159 | 7 | 125832718 | 125833851 | 4.17E-01 | 0.2132 | 0.0296 |
| CNP1179 | 7 | 142155609 | 142167486 | 4.18E-01 | 0.0537 | 0.0160 |
| CNP11703 | 10 | 32290286 | 32306952 | 4.22E-01 | 0.9890 | 0.0433 |
| CNP10824 | 4 | 157188133 | 157192659 | 4.25E-01 | 0.9840 | 0.0350 |
| CNP2007 | 14 | 40680246 | 40727099 | 4.31E-01 | 0.7201 | 0.0403 |
| CNP12287 | 15 | 22161116 | 22202157 | 4.39E-01 | 0.9517 | 0.0403 |
| CNP2238 | 17 | 14984651 | 14995227 | 4.48E-01 | 0.6655 | 0.0099 |
| CNP10917 | 5 | 32142837 | 32194212 | 4.53E-01 | 0.9438 | 0.0198 |
| CNP1813 | 12 | 11113633 | 11132799 | 4.57E-01 | 0.5926 | 0.0448 |
| CNP12118 | 13 | 88594853 | 88637416 | 4.62E-01 | 0.9737 | 0.0053 |
| CNP11272 | 7 | 110824661 | 110829591 | 4.66E-01 | 0.9831 | 0.0464 |
| CNP2563 | 22 | 23993985 | 24248712 | 4.78E-01 | 0.9437 | 0.0357 |
| CNP109 | 1 | 150822330 | 150853218 | 5.03E-01 | 0.1866 | 0.0068 |
| CNP980 | 6 | 81341677 | 81346253 | 5.09E-01 | 0.6591 | 0.0061 |
| CNP1452 | 9 | 67675871 | 67678367 | 5.13E-01 | 0.2973 | 0.0479 |
| CNP360 | 3 | 205022 | 207800 | 5.29E-01 | 0.3899 | 0.0220 |
| CNP573 | 4 | 32122898 | 32126696 | 5.30E-01 | 0.2074 | 0.0296 |
| CNP769 | 5 | 17563468 | 17568801 | 5.33E-01 | 0.9875 | 0.0471 |
| CNP1924 | 13 | 36970036 | 36982757 | 5.42E-01 | 0.6251 | 0.0228 |
| CNP975 | 6 | 77496587 | 77509523 | 5.46E-01 | 0.9526 | 0.0190 |
| CNP11196 | 7 | 16315368 | 16368639 | 5.53E-01 | 0.9808 | 0.0076 |
| CNP11487 | 8 | 86726773 | 86744665 | 5.57E-01 | 0.9899 | 0.0160 |
| CNP10426 | 2 | 194295401 | 194306617 | 5.58E-01 | 0.9655 | 0.0426 |
| CNP493 | 3 | 150446084 | 150450811 | 5.66E-01 | 0.8820 | 0.0068 |
| CNP10922 | 5 | 41623028 | 41630861 | 5.81E-01 | 0.9737 | 0.0372 |
| CNP1861 | 12 | 69158942 | 69164294 | 5.82E-01 | 0.5025 | 0.0213 |
| CNP12257 | 15 | 18415652 | 18454752 | 5.92E-01 | 0.9332 | 0.0053 |
| CNP296 | 2 | 146580874 | 146583404 | 6.00E-01 | 0.0668 | 0.0433 |
| CNP10849 | 4 | 178443731 | 178452258 | 6.02E-01 | 0.9864 | 0.0160 |
| CNP104 | 1 | 147303148 | 147526040 | 6.06E-01 | 0.7712 | 0.0494 |
| CNP10744 | 4 | 78495579 | 78500367 | 6.10E-01 | 0.9447 | 0.0365 |
| CNP257 | 2 | 89636367 | 89912071 | 6.22E-01 | 0.8950 | 0.0038 |
| CNP363 | 3 | 1658250 | 1666567 | 6.28E-01 | 0.9394 | 0.0243 |
| CNP12091 | 13 | 63227094 | 63303323 | 6.34E-01 | 0.9770 | 0.0182 |
| CNP958 | 6 | 66456258 | 66460573 | 6.41E-01 | 0.4760 | 0.0144 |
| CNP1956 | 13 | 71375556 | 71378557 | 6.42E-01 | 0.8197 | 0.0319 |
| CNP2063 | 15 | 22276386 | 22297003 | 6.43E-01 | 0.9665 | 0.0068 |
| CNP11171 | 6 | 169249708 | 169260864 | 6.44E-01 | 0.9220 | 0.0023 |
| CNP2603 | 23 | 13386706 | 13392612 | 6.51E-01 | 0.4956 | 0.0296 |
| CNP11914 | 11 | 93337704 | 93341240 | 6.56E-01 | 0.8743 | 0.0357 |
| CNP12162 | 14 | 41936937 | 42030401 | 6.68E-01 | 0.9878 | 0.0167 |
| CNP1679 | 11 | 5744656 | 5765715 | 6.80E-01 | 0.6055 | 0.0486 |
| CNP11826 | 11 | 5842285 | 5892086 | 6.93E-01 | 0.9359 | 0.0471 |
| CNP1620 | 10 | 66977929 | 66984452 | 7.05E-01 | 0.3219 | 0.0122 |
| CNP992 | 6 | 103844669 | 103868754 | 7.08E-01 | 0.1237 | 0.0129 |
| CNP575 | 4 | 34455420 | 34500578 | 7.08E-01 | 0.8343 | 0.0008 |
| CNP11142 | 6 | 140429446 | 140432237 | 7.10E-01 | 0.9433 | 0.0296 |
| CNP2733 | 24 | 23920276 | 26847931 | 7.18E-01 | 0.2232 | 0.0190 |
| CNP1346 | 8 | 115703663 | 115710907 | 7.20E-01 | 0.7039 | 0.0220 |
| CNP10456 | 2 | 242669783 | 242683192 | 7.20E-01 | 0.9782 | 0.0312 |
| CNP2639 | 23 | 63642592 | 63651585 | 7.21E-01 | 0.4802 | 0.0395 |
| CNP12610 | 18 | 65358832 | 65368255 | 7.21E-01 | 0.9710 | 0.0281 |
| CNP10310 | 2 | 77830206 | 77850174 | 7.33E-01 | 0.9643 | 0.0342 |
| CNP11655 | 9 | 114894172 | 114923574 | 7.35E-01 | 0.9652 | 0.0251 |
| CNP79 | 1 | 105820728 | 105823898 | 7.39E-01 | 0.1817 | 0.0312 |
| CNP11533 | 9 | 139481 | 264606 | 7.41E-01 | 0.9857 | 0.0099 |
| CNP2707 | 23 | 148692078 | 148789418 | 7.44E-01 | 0.5039 | 0.0015 |
| CNP2184 | 16 | 54353890 | 54379945 | 7.50E-01 | 0.3384 | 0.0175 |
| CNP2682 | 23 | 118919168 | 118941320 | 7.52E-01 | 0.5021 | 0.0068 |
| CNP11298 | 7 | 139650508 | 139654935 | 7.60E-01 | 0.9691 | 0.0175 |
| CNP110 | 1 | 151028547 | 151035324 | 7.75E-01 | 0.9406 | 0.0296 |
| CNP1175 | 7 | 141693868 | 141712586 | 7.80E-01 | 0.1142 | 0.0023 |
| CNP2118 | 15 | 82331742 | 82334554 | 7.80E-01 | 0.3432 | 0.0471 |
| CNP1726 | 11 | 49716131 | 49717264 | 7.81E-01 | 0.6987 | 0.0388 |
| CNP969 | 6 | 74648953 | 74658138 | 7.89E-01 | 0.4390 | 0.0152 |
| CNP2706 | 23 | 148452463 | 148462404 | 7.95E-01 | 0.4880 | 0.0342 |
| CNP2560 | 22 | 22680529 | 22726814 | 7.96E-01 | 0.1060 | 0.0205 |
| CNP874 | 5 | 150185693 | 150198797 | 7.99E-01 | 0.3038 | 0.0213 |
| CNP1922 | 13 | 31430622 | 31436423 | 8.03E-01 | 0.5726 | 0.0251 |
| CNP10549 | 3 | 89477282 | 89502071 | 8.04E-01 | 0.9820 | 0.0243 |
| CNP2406 | 19 | 40541333 | 40553688 | 8.09E-01 | 0.5593 | 0.0266 |
| CNP12439 | 16 | 34879419 | 34916829 | 8.14E-01 | 0.9850 | 0.0106 |
| CNP1236 | 8 | 5586134 | 5591735 | 8.17E-01 | 0.9011 | 0.0068 |
| CNP10815 | 4 | 153209736 | 153212191 | 8.22E-01 | 0.8515 | 0.0099 |
| CNP12062 | 13 | 22443593 | 22451522 | 8.28E-01 | 0.9448 | 0.0426 |
| CNP1607 | 10 | 58572176 | 58606915 | 8.33E-01 | 0.8995 | 0.0084 |
| CNP124 | 1 | 173063179 | 173068463 | 8.36E-01 | 0.5288 | 0.0076 |
| CNP11937 | 11 | 134119190 | 134132097 | 8.38E-01 | 0.9802 | 0.0403 |
| CNP1269 | 8 | 15447431 | 15455979 | 8.38E-01 | 0.8922 | 0.0205 |
| CNP12152 | 14 | 25832761 | 25849239 | 8.42E-01 | 0.9898 | 0.0289 |
| CNP11591 | 9 | 37494703 | 37504032 | 8.44E-01 | 0.9554 | 0.0365 |
| CNP766 | 5 | 15772218 | 15773597 | 8.44E-01 | 0.8819 | 0.0418 |
| CNP555 | 4 | 10001452 | 10009766 | 8.50E-01 | 0.7473 | 0.0030 |
| CNP11331 | 7 | 153130910 | 153299551 | 8.51E-01 | 0.9830 | 0.0046 |
| CNP638 | 4 | 98391437 | 98404155 | 8.51E-01 | 0.8296 | 0.0418 |
| CNP2419 | 19 | 48248681 | 48288811 | 8.56E-01 | 0.9728 | 0.0160 |
| CNP2648 | 23 | 76053855 | 76057477 | 8.62E-01 | 0.4829 | 0.0319 |
| CNP413 | 3 | 53003415 | 53016559 | 8.67E-01 | 0.9047 | 0.0319 |
| CNP11623 | 9 | 71288388 | 71309940 | 8.71E-01 | 0.9813 | 0.0167 |
| CNP2453 | 20 | 1505219 | 1508562 | 8.73E-01 | 0.9897 | 0.0456 |
| CNP10359 | 2 | 110173879 | 110519409 | 8.74E-01 | 0.9647 | 0.0198 |
| CNP1941 | 13 | 56656271 | 56676381 | 8.78E-01 | 0.6750 | 0.0053 |
| CNP1828 | 12 | 27986760 | 27988911 | 8.93E-01 | 0.7247 | 0.0099 |
| CNP2704 | 23 | 146651390 | 146655904 | 8.95E-01 | 0.4986 | 0.0099 |
| CNP2736 | 24 | 24720477 | 25412124 | 9.05E-01 | 0.1405 | 0.0152 |
| CNP2654 | 23 | 81283429 | 81298800 | 9.16E-01 | 0.5007 | 0.0068 |
| CNP148 | 1 | 195089940 | 195168372 | 9.19E-01 | 0.9536 | 0.0091 |
| CNP10303 | 2 | 73729307 | 73782394 | 9.21E-01 | 0.9778 | 0.0122 |
| CNP2675 | 23 | 109825336 | 109826953 | 9.38E-01 | 0.4870 | 0.0243 |
| CNP12744 | 20 | 59003765 | 59023478 | 9.39E-01 | 0.9877 | 0.0236 |
| CNP2636 | 23 | 62374362 | 62422551 | 9.43E-01 | 0.5004 | 0.0076 |
| CNP12679 | 19 | 58019782 | 58045481 | 9.44E-01 | 0.9327 | 0.0274 |
| CNP11737 | 10 | 58516320 | 58519894 | 9.45E-01 | 0.8983 | 0.0319 |
| CNP2619 | 23 | 35540930 | 35543706 | 9.50E-01 | 0.4868 | 0.0380 |
| CNP11949 | 12 | 7895692 | 8015167 | 9.50E-01 | 0.9440 | 0.0342 |
| CNP876 | 5 | 151495579 | 151498544 | 9.51E-01 | 0.9570 | 0.0152 |
| CNP10218 | 1 | 232770219 | 232774658 | 9.53E-01 | 0.9803 | 0.0357 |
| CNP10863 | 4 | 188099187 | 188115497 | 9.54E-01 | 0.9871 | 0.0129 |
| CNP770 | 5 | 17644656 | 17698273 | 9.57E-01 | 0.9747 | 0.0258 |
| CNP2621 | 23 | 47765101 | 47865818 | 9.59E-01 | 0.4915 | 0.0038 |
| CNP2439 | 19 | 59989695 | 60018425 | 9.61E-01 | 0.9472 | 0.0274 |
| CNP2062 | 15 | 22226226 | 22269689 | 9.62E-01 | 0.5589 | 0.0426 |
| CNP2422 | 19 | 48394873 | 48448077 | 9.70E-01 | 0.3481 | 0.0312 |
| CNP2604 | 23 | 16382145 | 16387877 | 9.71E-01 | 0.5046 | 0.0000 |
| CNP1447 | 9 | 69168903 | 69213467 | 9.79E-01 | 0.9899 | 0.0182 |
| CNP216 | 2 | 41091947 | 41099391 | 9.79E-01 | 0.9019 | 0.0243 |
| CNP2417 | 19 | 47986230 | 48149894 | 9.80E-01 | 0.9162 | 0.0129 |
| CNP12061 | 13 | 21994827 | 22004855 | 9.92E-01 | 0.9685 | 0.0395 |

Note:

1. AF: allele frequency (the total proportion of subjects with copy number less or more than two in total samples);

2. CC: uncertain or missing copy calls of CNPs;

1. The NCBI reference genome is Bulid 36.1.
