# Supplementary material for: Copy Number Variation in CNP267 Region May Be Associated with Hip Bone Size
Source: PLoS One. 2011 Jul 15;6(7):e22035. doi: 10.1371/journal.pone.0022035 (PMC3137628; doi:10.1371/journal.pone.0022035)
Supplement: Table S2 — Candidate genes at the interesting CNP regions in initial Chinese sample. (DOC) [file pone.0022035.s002.doc]

**Table S2 Candidate genes at the interesting CNP regions in initial Chinese sample**

| NAME | Chr | Start | End | P value | AF | CC | Candidate Genes |
| --- | --- | --- | --- | --- | --- | --- | --- |
| CNP10799 | 4 | 138543995 | 138549443 | 7.23E-03 | 0.0103 | 0.0478 | LOC641364 |
| CNP11164 | 6 | 162658558 | 162660430 | 6.18E-04 | 0.1081 | 0.0441 | PARK2,LOC100129958 |
| CNP182 | 1 | 246815817 | 246863836 | 1.05E-03 | 0.3055 | 0.003 | CNST,SCCPDH |
| CNP1196 | 7 | 154024104 | 154031766 | 0.013 | 0.9088 | 0.0387 | DPP6 |
| CNP1222 | 8 | 3774468 | 3777514 | 0.019 | 0.0858 | 0.0304 | CSMD1,LOC100421027 |
| CNP930 | 6 | 31394255 | 31404430 | 0.033 | 0.807 | 0.0281 | MICA,RPL15P4 |
| CNP168 | 1 | 227883479 | 227886819 | 0.038 | 0.7177 | 0.0312 | ZNF847P,LOC100130093 |
| CNP1811 | 12 | 9524645 | 9619559 | 0.043 | 0.6811 | 0.0015 | LOC728715,OVOS, DDX12, LOC100419520 |
| CNP12616 | 18 | 74763700 | 74765854 | 0.046 | 0.0191 | 0.0433 | MBP,GALR1 |

Note:

1. AF: allele frequency (the total proportion of subjects with copy number less or more than two in total samples);

2. CC: uncertain or missing copy calls of CNPs;

1. The NCBI reference genome is Bulid 36.1.
